# Inhibition of late sodium current via PI3K/Akt signaling prevents cellular remodeling in tachypacing-induced HL-1 atrial myocytes

**DOI:** 10.1007/s00424-022-02754-z

**Published:** 2022-10-24

**Authors:** Tae Hee Ko, Daun Jeong, Byeongil Yu, Ji Eun Song, Qui Anh Le, Sun-Hee Woo, Jong-Il Choi

**Affiliations:** 1 Division of Cardiology, Department of Internal Medicine, Korea University College of Medicine and Korea University Medical Centre, 73, Goryeodae-ro, Seongbuk-gu, Seoul, 02841 Republic of Korea; 2 Ion Channel Research Unit, Cardiovascular Research Institute, Korea University, Seoul, Republic of Korea; 3 Laboratory of Pathophysiology, College of Pharmacy, Chungnam National University, 99 Daehak-ro, Yuseong-gu, Daejeon, 34134 Republic of Korea

**Keywords:** Late sodium current, Atrial Fibrillation, Electrical remodeling, Tachypacing, PI3K/Akt signaling

## Abstract

An aberrant late sodium current (*I*_Na,Late_) caused by a mutation in the cardiac sodium channel (Na_v_1.5) has emerged as a contributor to electrical remodeling that causes susceptibility to atrial fibrillation (AF). Although downregulation of phosphoinositide 3-kinase (PI3K)/Akt signaling is associated with AF, the molecular mechanisms underlying the negative regulation of *I*_Na,Late_ in AF remain unclear, and potential therapeutic approaches are needed. In this work, we constructed a tachypacing-induced cellular model of AF by exposing HL-1 myocytes to rapid electrical stimulation (1.5 V/cm, 4 ms, 10 Hz) for 6 h. Then, we gathered data using confocal Ca^2+^ imaging, immunofluorescence, patch-clamp recordings, and immunoblots. The tachypacing cells displayed irregular Ca^2+^ release, delayed afterdepolarization, prolonged action potential duration, and reduced PI3K/Akt signaling compared with controls. Those detrimental effects were related to increased *I*_Na,Late_ and were significantly mediated by treatment with the *I*_Na,Late_ blocker ranolazine. Furthermore, decreased PI3K/Akt signaling via PI3K inhibition increased *I*_Na,Late_ and subsequent aberrant myocyte excitability, which were abolished by *I*_Na,Late_ inhibition, suggesting that PI3K/Akt signaling is responsible for regulating pathogenic *I*_Na,Late_. These results indicate that PI3K/Akt signaling is critical for regulating *I*_Na,Late_ and electrical remodeling, supporting the use of PI3K/Akt-mediated *I*_Na,Late_ as a therapeutic target for AF.

## Introduction

Atrial fibrillation (AF) is a common heart arrhythmia characterized by structural and electrical remodeling that leads to cardiac diseases such as heart failure [2, 7, 18]. Increasing evidence suggests that ion channel regulation is an effective therapeutic target for AF [8, 14]. Although ion channel-targeting drugs are available, the mechanism underlying those therapies remains to be determined.

The cardiac sodium channel (Na_v_1.5) plays a pivotal role in the initiation and propagation of action potential (AP) via membrane depolarization [22]. Mutations in Na_v_1.5 are mainly caused by aberrant sodium currents (*I*_Na_) that lead to arrhythmogenesis and produce conditions such as long QT syndrome, Brugada syndrome, and AF [30]. Those arrhythmic events are mainly caused by abnormal inactivation of Na_v_1.5, called “late *I*_Na_” (*I*_Na,Late_) [25]. Importantly, an increase in *I*_Na,Late_ can affect myocyte excitability through abnormal Na_v_1.5 inactivation, thereby inducing proarrhythmic events such as AF [32]. Thus, *I*_Na,Late_ regulation could be a potential target for AF treatment.

Recent studies revealed that phosphoinositide-3-kinase (PI3K) can be an effective treatment for arrhythmic events [33]. PI3K is a heterodimeric protein with catalytic subunits, such as p110α, that play crucial roles in cell survival, growth, and proliferation. Its downstream molecule, Akt, is involved in the regulation of Na_v_1.5 [3, 29]. A previous report revealed that PI3K inhibition caused the prolongation of AP duration (APD) and increased *I*_Na,Late_ in diabetic mouse hearts [20]. Moreover, mouse hearts lacking PI3K p110ɑ showed longer APD caused by increasing *I*_Na,Late_ [21]. However, the underlying mechanisms of PI3K/Akt signaling that regulate *I*_Na,Late_ following the development of AF remain unclear. In this work, we investigated the effects of PI3K/Akt signaling and its potential roles in *I*_Na,Late_ and AF using tachypacing-induced HL-1 myocytes.

## Materials and methods

### HL-1 atrial cell line culture and rapid electrical pacing

The HL-1 atrial myocyte cell line used (Merck Millipore) was derived from the AT-1 mouse atrial carcinoma cell line [9]. Cells were cultured on gelatin-fibronectin-coated culture dishes with Claycomb medium (Sigma) supplemented with 10% fetal bovine serum (Merck), 10 μM norepinephrine (Sigma), 2 mM l-glutamine (Sigma), and 1% penicillin/streptomycin (Gendepot) in a 37 °C, 5% CO_2_ atmosphere. To induce tachypacing, HL-1 atrial myocytes were subjected to rapid electrical pacing for 6 h at 1.5 V/cm, 4 ms, and 10 Hz in a 37 °C, 5% CO_2_ incubator. Cells not treated with pacing for 6 h were used as baseline controls.

### Drug application

Ranolazine (Abcam, CAS #. 142,387–99-3) was dissolved in dimethyl sulfoxide (DMSO, Biosesang) and applied to cells at a final concentration of 50 μM for 2 h before pacing was terminated. PI-103, a PI3K inhibitor, was dissolved in DMSO (Biosesang) and applied to cells at a final concentration of 100 nM for 2 h in the presence or absence of ranolazine. MK-2206, an Akt inhibitor, was dissolved in DMSO and applied to cells at a final concentration of 1 μM for 2 h. The maximum concentration of DMSO in the culture medium was 0.2%.

### Cell viability assay

Cell viability was analyzed via trypan blue exclusion. Cells were trypsinized and resuspended in culture medium. The cell suspension was mixed with the same volume of 0.4% trypan blue solution (Gendepot) and incubated for 1 min. Cell viability was determined using a C-Chip hemocytometer (Incyto) by counting the number of live and dead cells under a light microscope (Olympus).

### Electrophysiology

APs and Na^+^ currents (*I*_Na_) were recorded from HL-1 cells placed onto the recording chamber of a microscope (Nikon Eclipse Ti2), and an Axopatch 200B amplifier (Axon Instrument) was used for voltage and current clamping at room temperature (23 ± 1 °C). HL-1 cells were placed in a chamber mounted on an inverted microscope. Patch pipettes were pulled from thin-walled borosilicate capillaries (Clark Electromedical Instruments) using a P-97 Flaming/Brown Micropipette Puller (Sutter Instrument Company), and the resistance was 3–5 MΩ when pipettes were filled with pipette solution. Seals were created using negative pressure to form a whole-cell configuration with a giga seal. All recordings were made within 5 min after the formation of the whole-cell configuration. The voltage and current signals were filtered using a 4-pole Bessel-type low-pass filter at 10 kHz and sampled at a rate of 25 kHz. Cell capacitance (pF) and access resistance (MΩ) were automatically calculated and used to compensate for capacitive current and normalize ion current (pA/pF). Data acquisition and analysis were performed using digitizers (DigiData 1550B) and analysis software pClamp 10.7 (Molecular Devices). Tetrodotoxin (TTX, 10 μM) was used to block voltage-gated Na^+^ channels.

HL-1 myocytes were placed in a chamber mounted on an inverted microscope, and AP measurements were made in the whole-cell configuration just described. We used K^+^-rich pipette filling solution containing (composition in mM) KCl 140, EGTA 5, glucose 5, HEPES 5, Mg-ATP 5, and MgCl_2_ 1 (pH 7.2 adjusted with KOH). Cells were continuously superfused with Normal Tyrode (NT) solution containing (composition in mM): NaCl 143, KCl 5.4, HEPES 5, NaH_2_PO_4_ 0.33, MgCl_2_ 0.5, CaCl_2_ 1.8, and glucose 10 (pH 7.4 adjusted with NaOH). Briefly, cells were stimulated with a 0.7 nA current pulse for 2 ms, and pulse trains were elicited at 0.5 Hz. Delayed afterdepolarizations (DADs) were counted more than two times from 10 consecutive APs and averaged by dividing by 20 s. AP amplitude, maximal upstroke velocity (dv/dt_max_), resting membrane potential, and repolarization of APD at 50% and 90% (APD_50_ and APD_90_) were analyzed from 10 consecutive APs using pClamp 10.7 software (Molecular Devices).

To record peak and late *I*_Na_ (*I*_Na,Peak_ and *I*_Na,Late_), cells were incubated in NT solution and switched to a tetraethylammonium (TEA) solution after being placed in the whole-cell configuration. The TEA solution contained (composition in mM) NaCl 120, TEA-Cl 20, KCl 5.4, CaCl_2_ 1.8, MgCl_2_ 1, HEPES 10, and glucose 10 (pH 7.4 adjusted with NaOH). Pipettes were filled with pipette solution containing (composition in mM) NaCl 10, CsCl 50, CsF 30, l-aspartic acid 50, EGTA 5, and HEPES 1 (pH 7.3 adjusted with CsOH). *I*_Na,Peak_ was elicited by 50-ms voltage steps at 5-mV increments to potentials ranging from − 100 to + 60 mV, with a holding potential of − 140 mV. *I*_Na,Late_ was elicited by 200-ms voltage steps in 10-mV increments to potentials ranging from − 120 to − 10 mV. The average was determined as between 50 and 150 ms and normalized to the peak current after subtracting the difference between that found in the presence and absence of TTX (10 μM) [12]. In some experiments, we added 1 μM phospholipids (all di-C8, Echelon Bioscences) in pipette solution. Voltage-dependent activation of *I*_Na_ was assessed by measuring the peak conductance (G), which was calculated as G = *I*_Na_ (V − V_rev_), where *I*_Na_ is the current for each voltage step (V), and V_rev_ is the reversal potential. Data were normalized by maximum peak conductance (G_max_) and fitted with the Boltzmann function, giving values for V_1/2_ and slope *k* where G/G_max_ = 1/[1 + exp ((V − V_1/2_)/*k*)]. The voltage dependence of steady-state inactivation curves was measured using standard two-pulse protocols from 120 mV of holding potential, and cells were held ranging from − 140 to + 20 mV for 500 ms (pre-pulse) and then subjected to a − 40-mV test pulse for 20 ms. Currents (I) were normalized to maximum I (I_max_), and curves were fitted by the Boltzmann function, giving values for the V_1/2_ and slope *k* where I/Imax = 1/[1 + exp ((V − V_1/2_)/*k*)].

### Confocal Ca^2+^ imaging

To measure intracellular Ca^2+^ signals, HL-1 myocytes were loaded with 3 μM fluo-4 AM for 30 min. The cells were continuously superfused with 1.8 mM Ca^2+^ containing NT solution composed of (in mM) 137 NaCl, 5.4 KCl, 10 HEPES, 1 MgCl_2_, and 10 glucose (pH of 7.4) at 36.5 °C. HL-1 cells grown to > 80% confluence normally show regular occurrences of Ca^2+^ transients in the absence of electrical stimulation [16]. Ca^2+^ fluorescence was imaged at 60 or 120 Hz in two dimensions using a laser scanning confocal imaging system (A1, Nikon) attached to an inverted microscope (Eclipse Ti, Nikon) fitted with a × 60 oil immersion objective lens (Plan Apo, Numerical Aperture 1.4, Nikon) [17]. Dyes were excited at 488 nm using an Ar laser (Ommichrome), and fluorescence emissions at > 510 nm were detected. Images were recorded and analyzed with workstation software, NIS Elements AR (v3.2, Nikon). To estimate Ca^2+^ increases, the average resting fluorescence intensity (F_0_) was calculated from several frames immediately before the Ca^2+^ upstroke. Tracings of local Ca^2+^ signals are shown as the average fluorescence of each region-of-interest normalized to the F_0_ (F/F_0_). The average F_0_, measured in normal HL-1 cells, was used to calculate the F/F_0_ and obtain Ca^2+^ traces in rapidly paced cells.

### Immunofluorescence

Cells grown on gelatin/fibronectin-coated coverslips were washed with phosphate-buffered saline (PBS) 2 times and fixed with 4% paraformaldehyde (Biosesang) for 20 min. The fixed cells were then blocked with PBS containing 1% bovine serum albumin (Gendepot) for 1 h. For intracellular proteins, 0.1% Triton X-100 was added in blocking buffer, and coverslips were incubated with primary antibodies (phosphorylated Akt from Cell signaling, PI3K p110ɑ (1:100, Abcam), and phosphorylated Akt (1:100, Cell Signaling) mixed in each blocking buffer using titers recommended in the antibody datasheets. The coverslips were then washed with 1 × PBS buffer 2 times and incubated with secondary antibodies labeled with Alexa Fluor 488 (Abcam). The coverslips were PBS-washed 2 times, counterstained with diamidino-2-phenylindole (Abcam), and mounted on slide glasses. Visualization of the slides was performed under a fluorescence microscope (Olympus). Total fluorescence intensity was measured and analyzed with ImageJ.

### Immunoblotting

The cells were washed with 1 × PBS 2 times and lysed in a Nonidet P-40 buffer (GenDepot) containing protease inhibitors (GenDepot) and a phosphatase inhibitor cocktail (GenDepot). The lysate was sonicated and incubated on ice for 5–15 min with intermittent vortexing, and then, the lysate was centrifuged at 14,000 rpm at 4 °C for 25 min. Supernatants were collected in clean microtubes. Total proteins were quantified via Bradford protein assay, and then, the proteins were size separated with acrylamide gel electrophoresis and transferred onto nitrocellulose membranes (Atto). The membranes were blocked for 1 h with 5% skim milk (Biosesang) in TBS-T (Biosesang) and incubated with primary antibodies overnight at 4 °C. The primary antibodies were as follows: Na_v_1.5 (1:200, Alomone), anti-PI3K p110ɑ (1:1000, Abcam), phosphorylated Akt (1:1000, cell signaling), Akt (1:1000, Abcam), and glyceraldehyde-3-phosphate dehydrogenase (GAPDH) antibody (1:5000, Enogene). We used horseradish peroxidase–conjugated secondary anti-rabbit goat antibody (Enzo) to detect blots with enhanced chemiluminescence solution (Thermo). The blots were detected and analyzed with Chemidoc (Bio-Rad) and Image Studio™ analysis software (LI-COR Biosciences).

### Experimental animals

Adult (10 weeks old) male C57BL/6 mice were used for this study. The mice were maintained in a specific pathogen-free animal facility at Korea Medical University under standardized conditions with 12-h light and dark cycles (light on from 8:00 am–8:00 pm) at 18–25 °C and free access to chow and water. All experimental procedures were performed in accordance with the Guide for the Care and Use of Laboratory Animals of the Republic of Korea. Experimental procedures were also approved by the Committee on Animal Research at Korea University College of Medicine.

### Electrocardiogram (ECG)

For in vivo ECGs, the mice were anesthetized by an intraperitoneal injection of Avertin (12.5 mg/ml; Sigma-Aldrich). The mice were kept on a heating pad (37 ± 0.5 °C) and baseline ECGs were recorded using four subcutaneous electrodes for lead II. For injections, PI-103 and ranolazine were dissolved in DMSO (Biosesang) and injected at a dose of 10 mg/kg and 20 mg/kg for 2 weeks, respectively. Control (CTRL) mice were injected with the same volume of DMSO. Surface ECGs were recorded at baseline, 1, and 2 weeks. ECG data recorded the RR interval, PR interval, QRS interval, and QT interval according to Bazett’s formula using the Powerlab acquisition system, and were analyzed using LabChart 8 Pro software (AD Instruments, Sydney, Australia).

### Statistical analysis

All experimental data are presented as the mean ± standard error of mean (SEM). For comparisons between groups, statistical differences were evaluated by one-way ANOVA with Tukey’s multiple comparison test using Prism 8 (GraphPad Software, La Jolla, CA, USA). *P*-values < 0.05 were considered to indicate significant differences between groups.

## Results

### *I*_*Na,Late*_ inhibition prevents myocyte remodeling in tachypacing-induced HL-1 myocytes

**Fig. 1:**
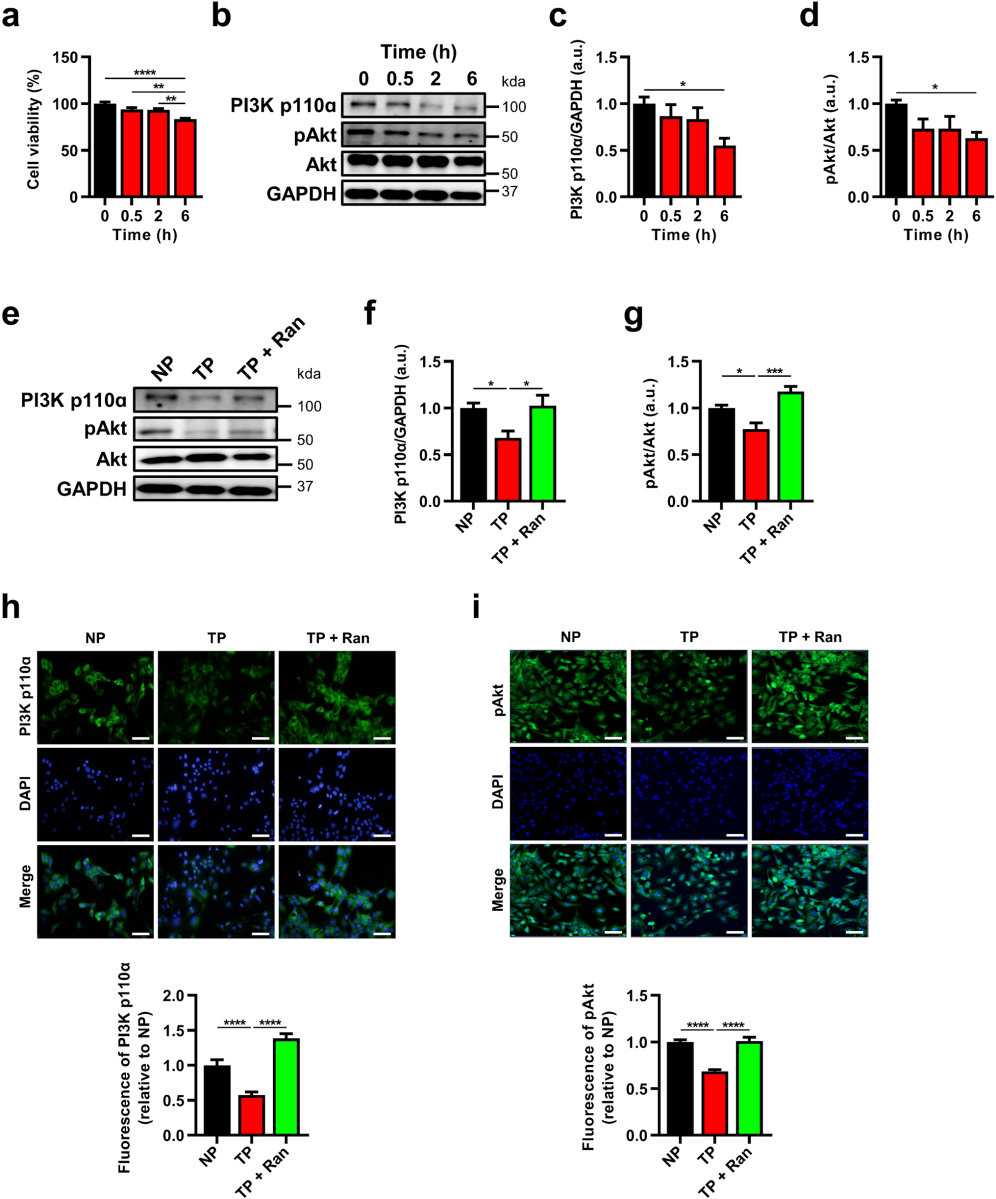
Effects of PI3K/Akt signaling on *I*_Na,Late_ inhibition in tachypacing-induced HL-1 myocytes. **a** Cell viability in tachypacing-induced HL-1 myocytes was analyzed in a time-dependent manner (*n* = 4 per group). **b** Representative immunoblot images of PI3K p110α and pAkt (*n* = 6 per group). **c**, **d** Quantified expression of PI3K p110ɑ and phosphorylated Akt (*n* = 6 per group). **e** Representative image of immunoblots: PI3K p110ɑ, phosphorylated Akt, total Akt, and GAPDH expression from HL-1 myocytes in the non-pacing (NP), rapid pacing (TP), and TP + ranolazine (TP + Ran) groups. **f**, **g** Quantitative protein levels of PI3K p110ɑ and phosphorylated Akt (*n* = 6 per group). **h** Representative immunofluorescence and quantified intensity of PI3K p110ɑ (*n* = 20 per group). **i** Immunofluorescence and quantified intensity of pAkt (*n* = 30 per group). Values are the mean ± SEM. ^*^p < 0.05, ^**^p < 0.01, ^***^p < 0.001, ^****^p < 0.0001. Comparison using one-way ANOVA followed by Tukey’s post hoc test

Rapid electrical stimulation is an established method for constructing AF models that has been used in many in vitro and in vivo studies [6]. In this study, HL-1 myocytes were exposed to rapid electrical stimulation to construct a cellular AF model. We found that 6-h tachypacing significantly lowered cell viability (Fig. 1a). Moreover, 6-h tachypacing lowered the expression of PI3K p110α and pAkt compared with 0-h tachypacing (Fig. 1b–d) and caused tachypacing-induced myocyte remodeling.

To assess whether decreased PI3K/Akt signaling could be reversed by *I*_*Na,Late*_ inhibition, we applied ranolazine (50 μM), an *I*_*Na,Late*_ blocker, for 2 h before the termination of tachypacing. We found that the reduction in PI3K p110α and pAkt expression induced by tachypacing was significantly recovered by *I*_*Na,Late*_ inhibition (Fig. 1e–g). Moreover, tachypacing lowered PI3K p110α and pAkt fluorescence, and *I*_*Na,Late*_ inhibition significantly reversed that trend in HL-1 myocytes (Fig. 1h, i). These results revealed that the potential effectiveness of *I*_*Na,Late*_ inhibition occurs via the upregulation of PI3K/Akt signaling in tachypacing-induced HL-1 myocytes.

### *I*_*Na,Late*_ inhibition recovers abnormal myocyte excitability in tachypacing-induced HL-1 myocytes

**Fig. 2:**
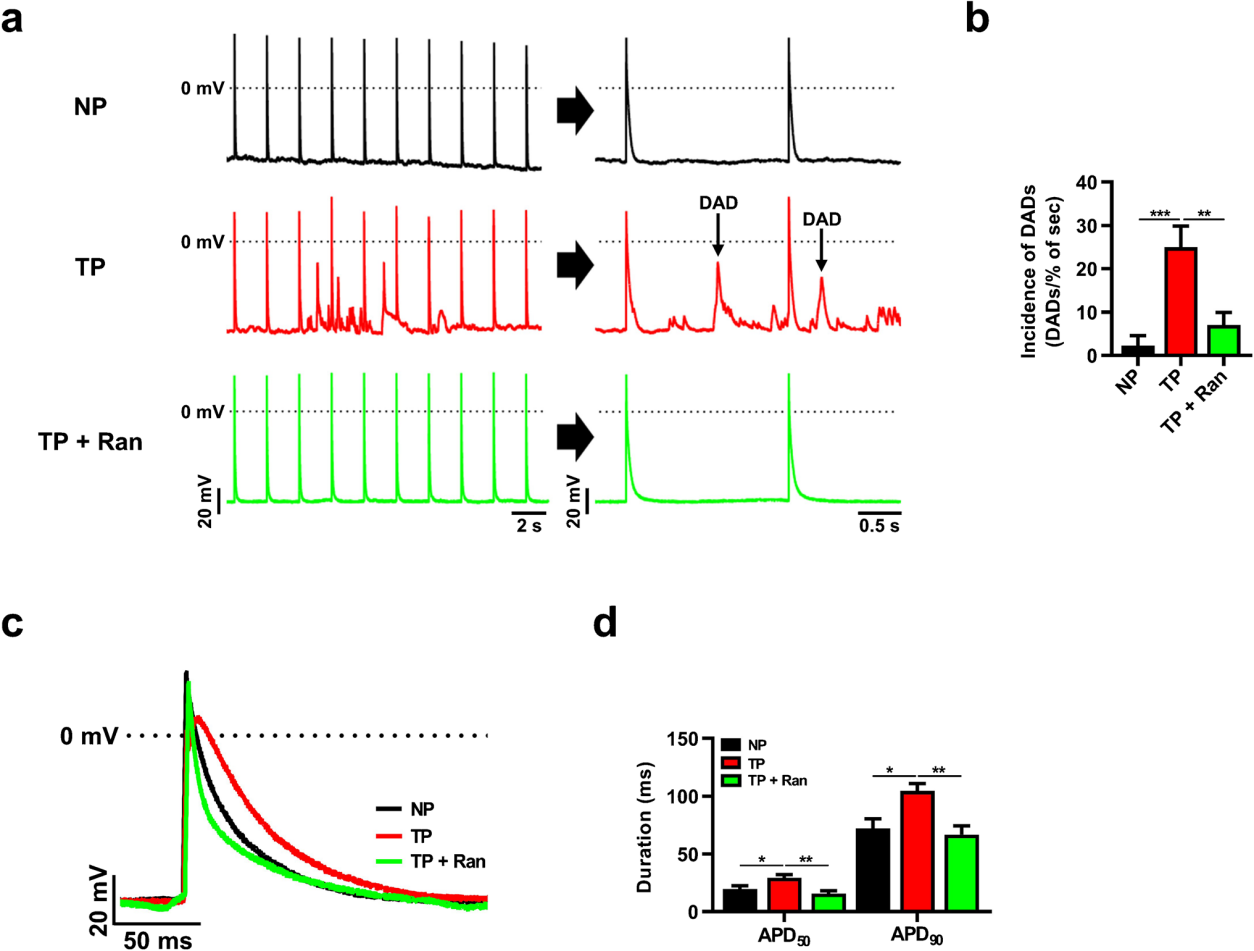
Effects of myocyte excitability on *I*_Na,Late_ inhibition in tachypacing-induced HL-1 myocytes. **a** Representative spontaneous AP trace (0.5 Hz) recorded from NP, TP, and TP + R HL-1 myocytes. **b** The occurrence of delayed afterdepolarizations (DADs) was analyzed from 10 consecutive APs in HL-1 myocytes. **c**, **d** Representative single AP traces and the repolarization of AP duration (APD) at 50% and 90% at 0.5 Hz. Values are the mean ± SEM. ^*^p < 0.05, ^**^p < 0.01, ^***^p < 0.001. Comparison using one-way ANOVA followed by Tukey’s post hoc test

The enhancement of *I*_*Na,Late*_ prolongs APD and induces triggered activity (early and delayed after depolarization) that can lead to severe arrhythmic disorders [5, 31]. To explore the effects of *I*_*Na,Late*_ inhibition on atrial excitability, we made AP recordings using the patch-clamp technique. Tachypacing caused severe DADs, and *I*_*Na,Late*_ inhibition markedly decreased DAD-induced arrhythmogenesis (Fig. 2a, b). Moreover, tachypacing significantly prolonged APD at 50% and 90% compared with non-paced HL-1 myocytes and that effect was markedly decreased by *I*_*Na,Late*_ inhibition (Fig. 2c, d). This observation supports the idea that *I*_*Na,Late*_ plays a major role in the atrial excitability of tachypacing-induced HL-1 myocytes.

### *I*_*Na,Late*_ inhibition suppresses abnormal* I*_*Na*_ properties in tachypacing-induced HL-1 myocytes

**Fig. 3:**
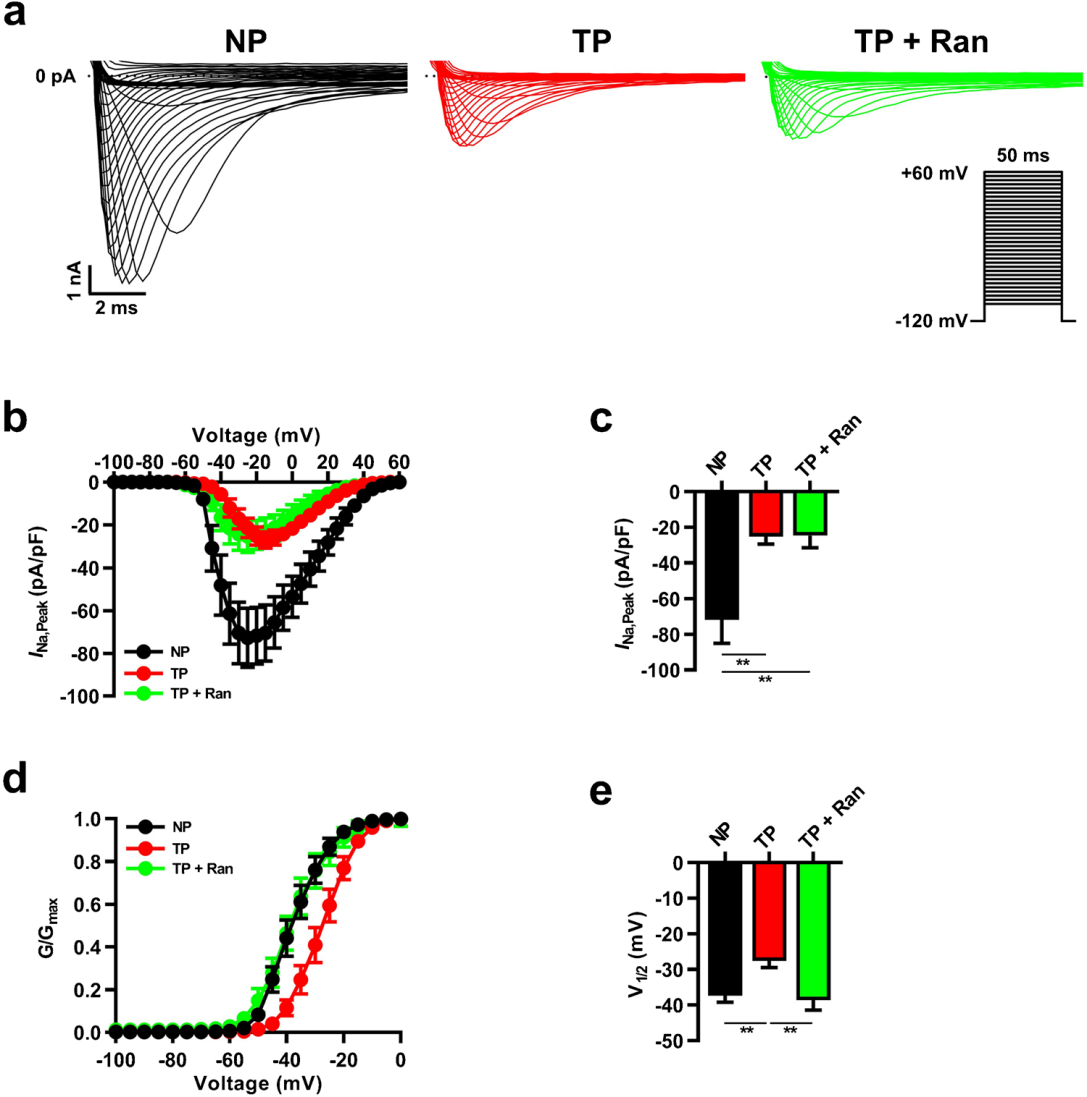
*I*_Na,Late_ inhibition affects the kinetics of *I*_Na_ activation in tachypacing-induced HL-1 myocytes. **a**, **b** Representative images of whole-cell *I*_Na,Peak_ and the current–voltage relationship (I-V curve) from NP, TP, and TP + Ran HL-1 atrial myocytes. **c** *I*_Na,Peak_ density was calculated at − 25 mV (*n* = 12–14 per group). **d**, **e** The voltage-dependent activation curve was determined by the Boltzmann function, and V_1/2_ was analyzed (*n* = 9–14 per group). Values are the mean ± SEM. ^**^p < 0.01. Comparison using one-way ANOVA followed by Tukey’s post hoc test

To investigate the effects of *I*_*Na,Late*_ inhibition on *I*_*Na*_ properties, we first recorded *I*_*Na,Peak*_ using the patch-clamp technique. As shown in Fig. 3a, whole-cell measurements of *I*_*Na,Peak*_ showed that tachypacing reduced *I*_*Na,Peak*_ density compared with non-paced HL-1 myocytes (Fig. 3b, c). *I*_*Na,Late*_ inhibition also decreased *I*_*Na,Peak*_ density compared with that in non-paced HL-1 myocytes, while *I*_*Na,Late*_-inhibited and tachypacing-induced HL-1 myocytes did not differ significantly from each other (Fig. 3b, c). However, tachypacing-induced cells displayed a more positive shift in *I*_*Na*_ activation than non-paced HL-1 myocytes and that change was significantly recovered by *I*_*Na,Late*_ inhibition (Fig. 3b, c), possibly by preventing the conduction defect [10].

**Fig. 4:**
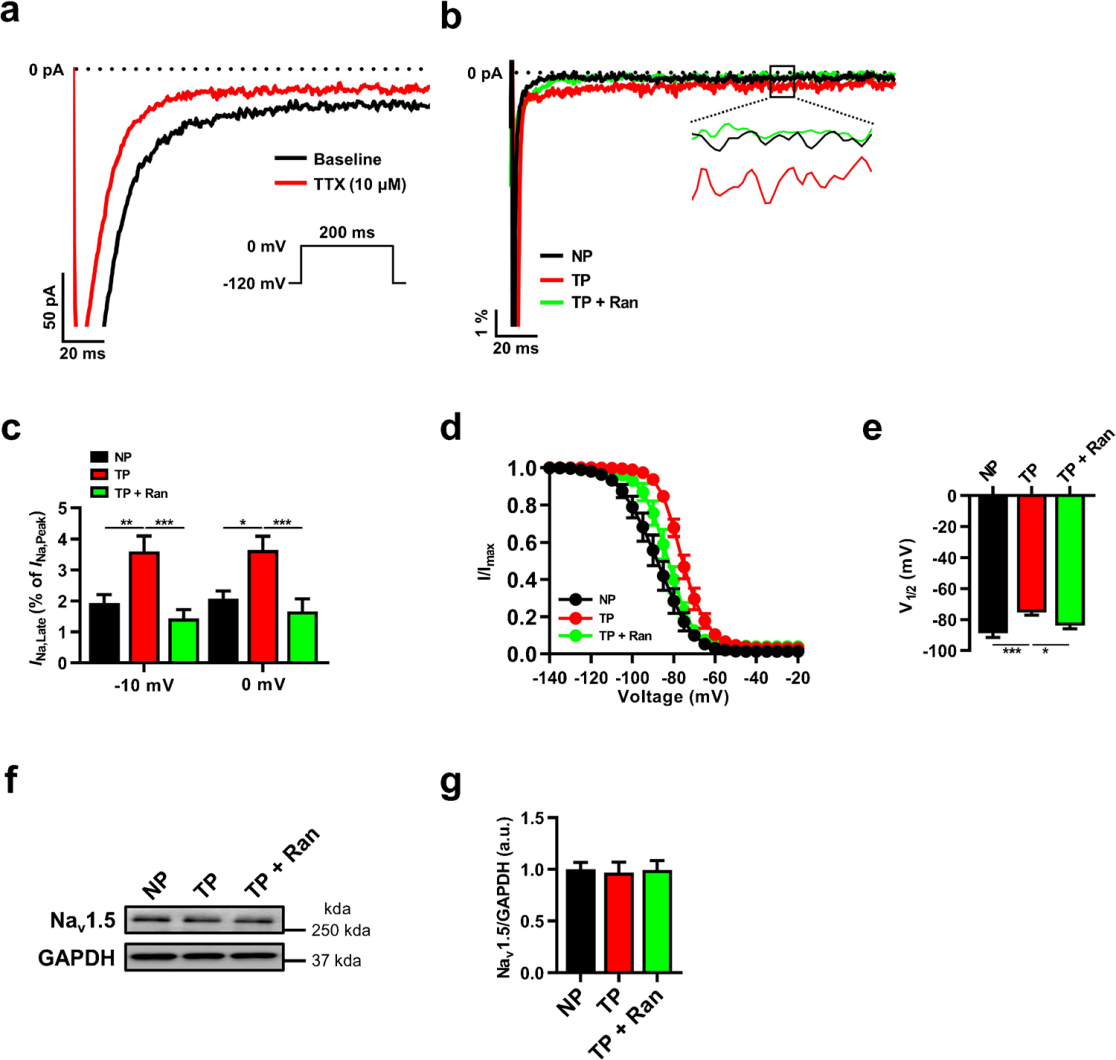
Dysregulated *I*_Na,Late_ is recovered by ranolazine treatment in tachypacing-induced HL-1 myocytes. **a** *I*_Na,Late_ was recorded after subtracting the difference measured in the presence and absence of TTX (10 μM) and elicited by a holding potential of − 120 mV from a 200-ms pulse at − 10 mV. **b**, **c** Representative traces of *I*_Na,Late_ are calculated as percentage of *I*_Na,Peak_ at − 10 and 0 mV (*n* = 10 per group). **d**, **e** The voltage-dependent steady-state inactivation curve was determined by the Boltzmann function, and V_1/2_ was analyzed (*n* = 9–11 per group). **f**, **g** Representative immunoblot image and analysis of Na_v_1.5 (*n* = 8 per group). Values are the mean ± SEM. ^*^p < 0.05, ^**^p < 0.01, ^***^p < 0.001. Comparison using one-way ANOVA followed by Tukey’s post hoc test

The regulation of *I*_*Na,Late*_ is crucial in the management of AF, although it is observed to ~ 1% of *I*_*Na,Late*_ [13]. To test its effects on pathological atrial remodeling, we next recorded *I*_*Na,Late*_ using a depolarizing voltage of − 10 mV (with − 140 mV of holding potential) and calculated normalized *I*_*Na,peak*_ between 50 and 150 ms, after subtracting the difference between that measured in the presence and absence of 10 μM TTX (Fig. 4a).

Those direct recordings showed that *I*_*Na,Late*_ was significantly higher in tachypacing-induced than non-paced HL-1 myocytes, and *I*_*Na,Late*_ inhibition markedly abolished that increase (Fig. 4b, c). Interestingly, tachypacing produced a positive shift in *I*_*Na*_ inactivation, which was markedly recovered by *I*_*Na,Late*_ inhibition (Fig. 4d, e), suggesting that *I*_*Na,Late*_ inhibition effectively reversed the aberrant kinetics of Na_v_1.5. Although we also detected Na_v_1.5 expression, we found no differences between groups (Fig. 4f, g). Our results revealed the potential role of *I*_*Na,Late*_ inhibition in *I*_*Na*_ open probability.

### Suppression of tachypacing-induced arrhythmic Ca^2+^ release by *I*_*Na,Late*_ inhibition

**Fig. 5:**
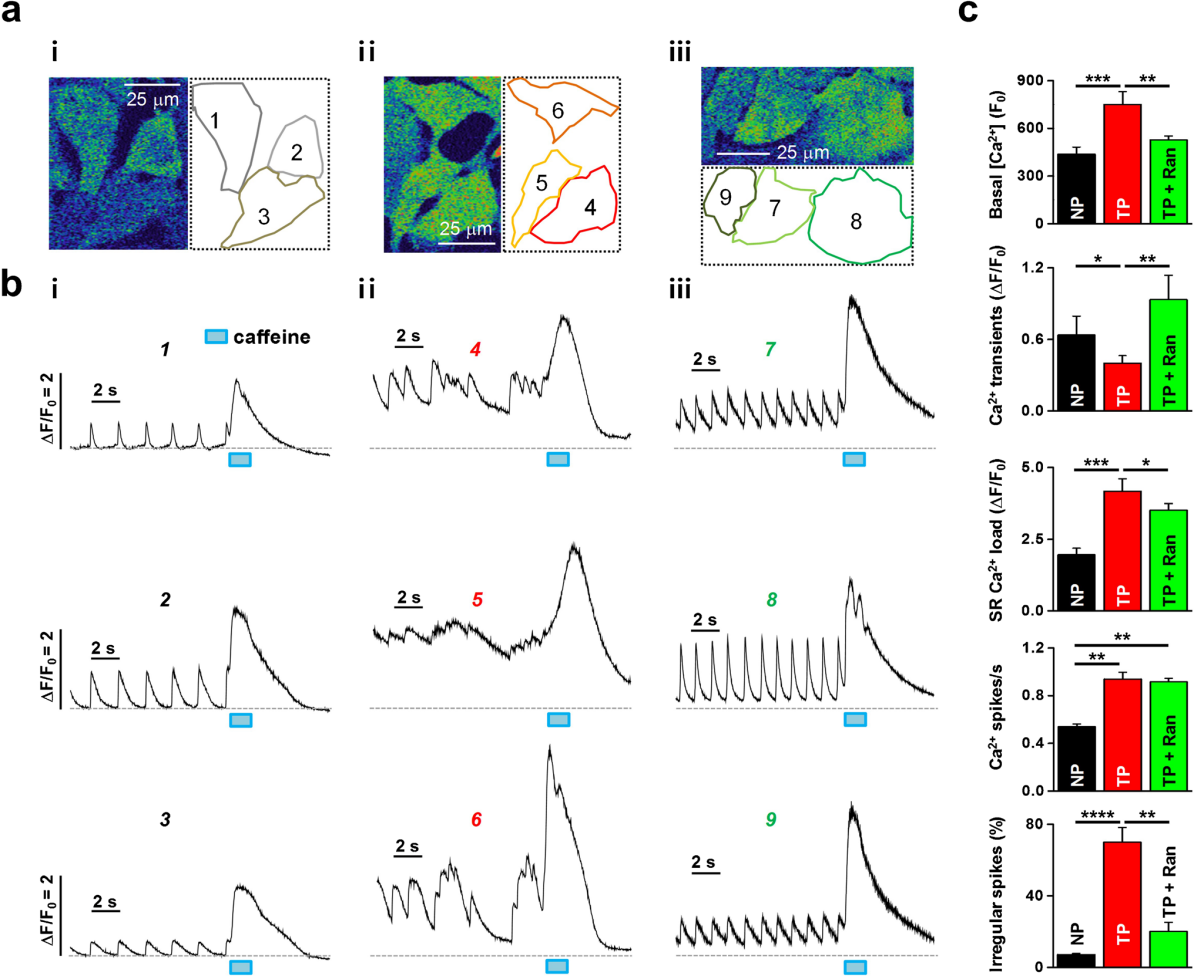
Disturbance of rhythmic Ca^2+^ signaling by tachypacing and its recovery by ranolazine in HL-1 myocytes. **a** Confocal image of representative HL-1 myocytes (labeled with the numbers 1–9) showing Ca^2+^ signals (left) and the regions-of-interest used to measure the Ca^2+^ signals (right). The confocal Ca^2+^ images were measured from normal (non-paced) HL-1 myocytes with a confluence of > 90% (i) and similarly confluent HL-1 myocytes subjected to tachypacing at 10 Hz for 6 h (ii) and tachypacing (10 Hz, 6 h) with a 2-h incubation in 50 μM ranolazine (iii). **b** Ca^2+^ traces measured from normal cells (i; 1–3) and tachypacing-induced HL-1 myocytes without (ii; 4–6) and with ranolazine (iii; 7–9), showing regular Ca^2+^ transients followed by caffeine (10 mM)-induced Ca^2+^ transients. **c** Comparisons of the basal Ca^2+^ level, Ca^2+^ transient magnitudes, the magnitude of caffeine-induced Ca^2+^ transients (SR Ca^2+^ load), the rate of Ca^2+^ transient occurrence (Ca^2+^ spikes/s), and the % of irregular Ca^2+^ spikes between normal cells (3 batches, 144 cells) and tachypacing-induced cells (10 Hz, 6 h) without (3 batches, 143 cells) and with (2 h, 10 μM; 3 batches, 128 cells) ranolazine. Values are the mean ± SEM. ^*^p < 0.05, ^**^p < 0.01, ^***^p < 0.001, ^****^p < 0.0001. Comparison using one-way ANOVA followed by Tukey’s post hoc test

*I*_Na,Late_ increases intracellular Na^+^, which can enhance the Ca^2+^ influx, ultimately inducing Ca^2+^ overload and electrical abnormality [4]. Thus, we next examined whether increases in *I*_Na,Late_ affected Ca^2+^ handling in tachypacing-induced HL-1 myocytes. Figure 5a (i) and b (i) (left panel) show representative Ca^2+^ traces measured from HL-1 myocytes under normal conditions. Those myocytes showed autorhythmic regular Ca^2+^ transients at a confluence of > 80% [16]. Caffeine (10 mM) was applied to those cells after we measured rhythmic Ca^2+^ releases to estimate the sarcoplasmic reticulum (SR) Ca^2+^ loading status. In HL-1 myocytes treated with 6 h of tachypacing, we observed irregular Ca^2+^ releases of varying magnitudes and higher basal Ca^2+^ levels (Fig. 5b (ii), c). In addition, we found more Ca^2+^ spikes than under control conditions. The average magnitude of Ca^2+^ transients in HL-1 myocytes with tachypacing was significantly smaller than that in HL-1 myocytes under control conditions (~ 60% of control levels; Fig. 5c). However, it should be noted that tachypacing-induced HL-1 myocytes had larger and more prolonged Ca^2+^ releases than control cells. Percentages of arrhythmic Ca^2+^ spikes relative to the number of regular Ca^2+^ peaks were estimated from Ca^2+^ traces recorded for 10 s. We found that the percentage of irregular Ca^2+^ transients was dramatically increased in tachypacing-induced HL-1 myocytes (Fig. 5b (ii), c). The amplitudes of caffeine-induced Ca^2+^ release increased by approximately twofold after 6 h of tachypacing (Fig. 5b (ii) and c), suggesting that the increase in SR Ca^2+^ load was caused by tachypacing.

We examined the effects of ranolazine (50 μM), which suppresses *I*_Na,Late_, on abnormal and arrhythmic Ca^2+^ signals in tachypacing-induced HL-1 myocytes. Ranolazine was added to the external solution for the last 2 h of the 6-h tachypacing period. Interestingly, most of the cells treated with ranolazine showed regular Ca^2+^ transients (Fig. 5b (iii), c). In addition, basal Ca^2+^ level was completely reversed to the control level in those cells. Nevertheless, the magnitude of Ca^2+^ transients, SR Ca^2+^ loading, and the rate of Ca^2+^ spiking remained higher than the control levels after the application of ranolazine, although SR Ca^2+^ loading was slightly reduced (Fig. 5b (iii), c). These results suggest that *I*_Na,Late_ could play an important role in the disruption of beating rhythm and basal Ca^2+^ increases in atrial myocytes under prolonged high-frequency electrical excitations that mimic AF. These data also suggest that *I*_Na,Late_ inhibition can reverse tachypacing-induced abnormalities in Ca^2+^ transients, maybe by suppressing Na^+^ influx-mediated Ca^2+^ increases. However, it should also be noted that the increased SR Ca^2+^ loading and higher beating rate induced by tachypacing might be only slightly attenuated by *I*_Na,Late_ inhibition.

### Dysregulation of myocyte excitability by PI3K inhibition is reversed by* I*_*Na,Late*_ inhibition

**Fig. 6:**
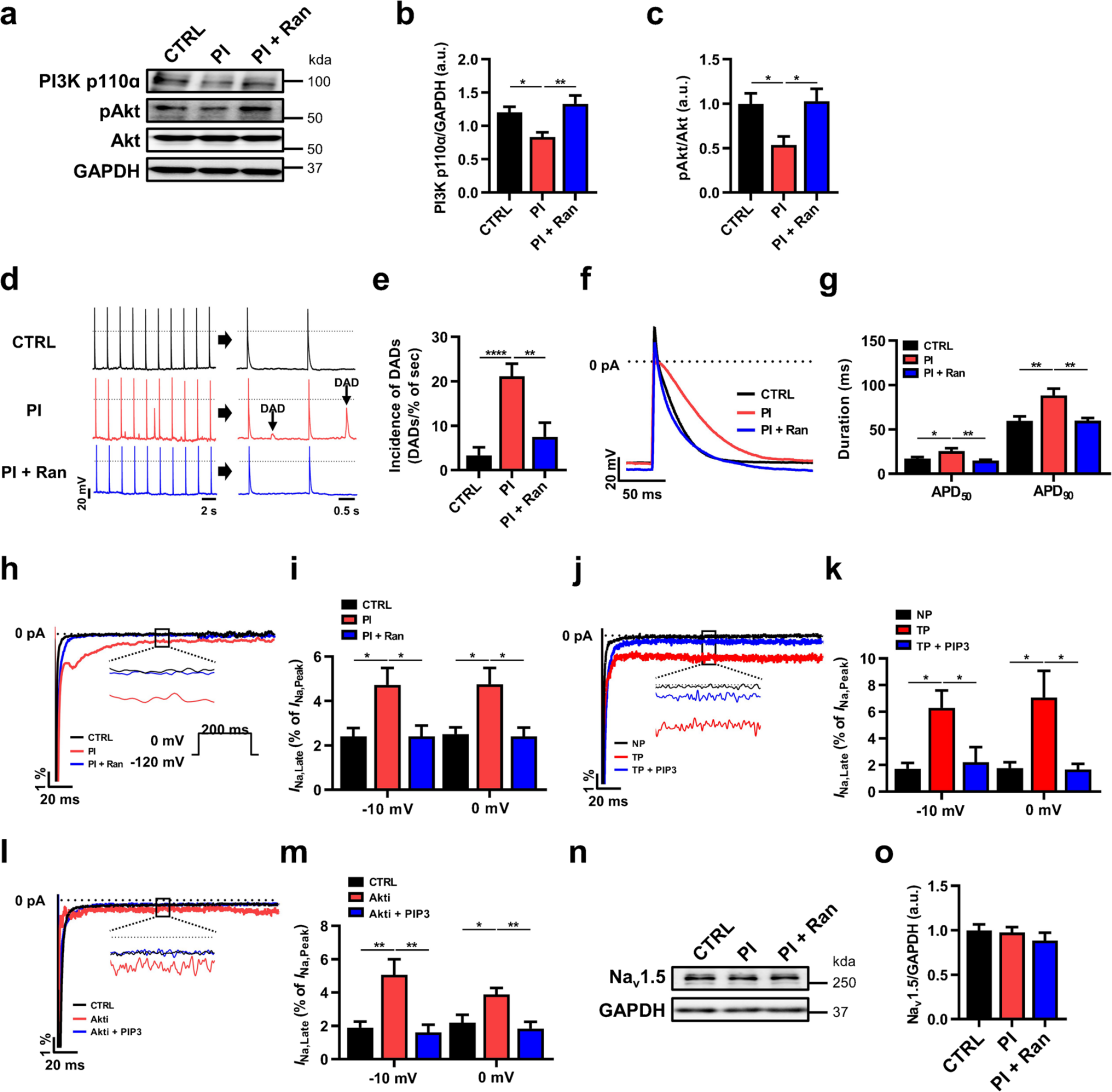
*I*_Na,Late_ is regulated by the PI3K/Akt pathway. **a**, **b** Representative traces of *I*_Na,Late_, calculated as a percentage of *I*_Na,Peak_ at − 10 or 0 mV (*n* = 7–9 per group). **c** Representative immunoblot images of PI3K p110α and pAkt from CTRL, PI, and PI + Ran groups of HL-1 myocytes (*n* = 6 per group). **d**, **e** Representative traces of APs and DADs were analyzed from CTRL, PI, and PI + Ran groups of HL-1 myocytes (*n* = 9–15 per group). **f**, **g** Representative single traces of APs and the repolarization of APD at 50% and 90% (*n* = 9–15 per group). **h**, **i** Representative traces of *I*_Na,Late_, calculated as a percentage of *I*_Na,Peak_ at − 10 or 0 mV (*n* = 7–9 per group). **j**, **k** Sample trace of *I*_Na,Late_ in non-pacing (NP), tachypacing (TP), and TP with or without infusion of PIP3 (*n* = 5–6 per group). **l**, **m** Representative images of *I*_Na,Late_ in control (CTRL), Akt inhibition (Akti), and Akti with or without infusion of PIP3 (*n* = 6 per group). **n**, **o** Representative immunoblot image and analysis for Na_v_1.5 (*n* = 8 per group). Values are the mean ± SEM. ^*^p < 0.05, ^**^p < 0.01, ^****^p < 0.0001. Comparison using one-way ANOVA followed by Tukey’s post hoc test

To determine whether PI3K inhibition impairs atrial excitability and further contributes to pathogenic *I*_Na,Late_, we measured the APs in HL-1 myocytes treated with PI-103 (100 nM) as a PI3K inhibitor or with co-application of PI-103 and ranolazine (50 μM) for 2 h and then divided the cells into three groups: control (CTRL), PI-103 treatment (PI), and co-application of PI-103 and ranolazine (PI + Ran). After PI3K inhibition, as shown in Fig. 6a, immunoblot analyses showed that PI3K p110ɑ and pAkt were significantly decreased compared with the CTRL group, and *I*_Na,Late_ inhibition completely reversed those changes to the CTRL level, demonstrating that *I*_Na,Late_ inhibition upregulated PI3K/Akt signaling (Fig. 6b, c).

To investigate the effects of atrial excitability on PI3K inhibition, we recorded APs using the patch-clamp technique. As shown in Fig. 6d, PI3K inhibition caused severe DADs, which was significantly abolished by *I*_Na,Late_ inhibition (Fig. 6e). Moreover, PI3K inhibition displayed prolonged APD_50_ and APD_90_ compared with control HL-1 myocytes (Fig. 6f, g), implying that PI3K inhibition had detrimental effects on atrial excitability. In contrast, *I*_Na,Late_ inhibition significantly reduced APD_50_ and APD_90_ compared with the PI-103-induced HL-1 myocytes (Fig. 6f, g). These results support the idea that PI3K/Akt signaling plays a pivotal role in *I*_Na,Late_-regulated myocyte excitability.

### PI3K/Akt is required for *I*_*Na,Late*_ regulation

Our findings open the possibility that PI3K/Akt signaling has an anti-arrhythmic effect on *I*_Na,Late_ regulation. Thus, we formulated our central hypothesis that PI3K/Akt signaling regulates pathogenic *I*_Na,Late_. To elucidate the direct implications of PI3K/Akt signaling for *I*_Na,Late_ regulation, we made direct recordings of *I*_Na,Late_. PI3K inhibition significantly increased *I*_Na,Late_ compared with control HL-1 myocytes and that increase was recovered by ranolazine (Fig. 6h, i). To test whether increasing *I*_Na,Late_ under pathological condition is associated with PI3K regulation, we recorded *I*_Na,Late_ in HL-1 myocytes with tachypacing or Akt inhibition by applying phosphatidylinositol 3,4,5-trisphosphate (PIP3), the second messenger produced by PI3K. Increasing *I*_Na,Late_ in tachypacing-induced HL-1 myocytes was recovered by infusion of PIP3 to non-pacing levels (Fig. 6j, k). Moreover, Akt inhibition also caused an increase in *I*_Na,Late_ but was significantly decreased by PIP3 infusion (Fig. 6l, m). Therefore, pathogenic *I*_Na,Late_ might be due to alterations in PI3K/Akt signaling. Although we performed immunoblotting of Na_v_1.5 expression, we found no significant difference between groups (Fig. 6n, o).

**Fig. 7:**
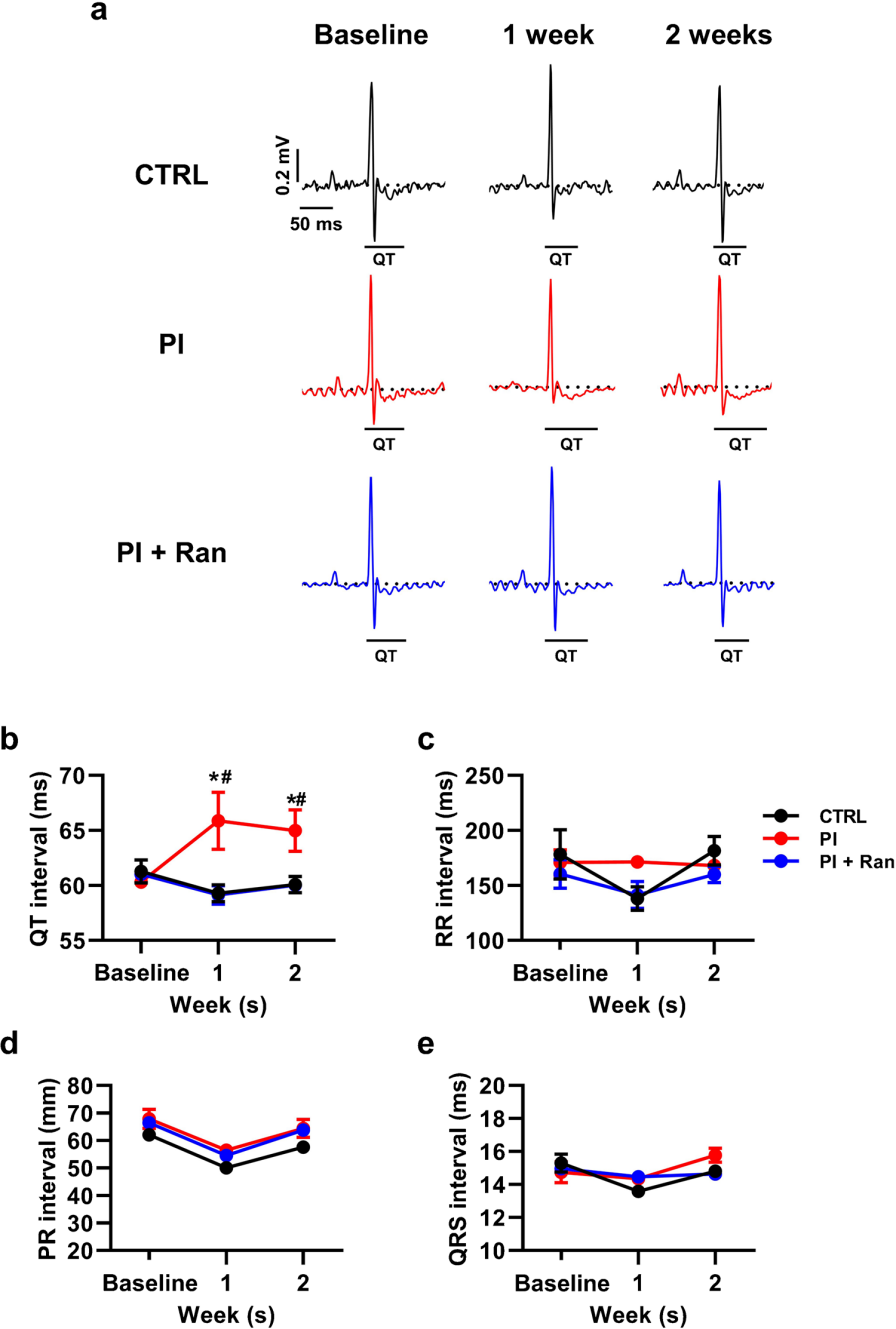
Prolongation of QT in PI3K inhibition is reversed by *I*_Na,Late_ inhibition in mouse heart. **a** Representative ECGs from CTRL (*n* = 6), PI (*n* = 6), and PI + Ran (*n* = 6) injected mice at pre-injection, 1, and 2 weeks. **b**–**e** Comparison of ECG parameters including QT, RR, PR, and QRS interval in CTRL, PI, and PI + Ran mice at baseline, 1, and 2 weeks. Values are the mean ± SEM. ^*^p < 0.05 vs. CTRL, ^#^*p* < 0.05 vs. PI + Ran. Comparison using one-way ANOVA followed by Tukey’s post hoc test

To elucidate the in vivo electrophysiological properties, we next recorded electrocardiograms (ECGs) from mice, which were divided into 3 groups: control (CTRL, DMSO injection), PI (PI-103 injection), and PI + Ran (PI-103 with ranolazine injection). We intraperitoneally injected PI-103 (10 mg/kg) alone or PI-103 with ranolazine (20 mg/kg) every day for 2 weeks. We found that PI3K inhibition displayed prolongation of QT interval, which was significantly shortened by *I*_Na,Late_ inhibition (Fig. 7a, b), leading to an anti-arrhythmic effect. However, there were no significant differences in the RR, PR, and QRS interval between groups (Fig. 7c–e). Our results imply that *I*_Na,Late_ inhibition is sufficient to alter electrophysiological properties and enhances PI3K/Akt signaling.

Taken together, our findings support the anti-arrhythmic effect of PI3K/Akt signaling on pathogenic *I*_Na,Late_ regulation, which prevents electrophysiological dysfunction and arrhythmic effects under AF conditions.

## Discussion

**Fig. 8:**
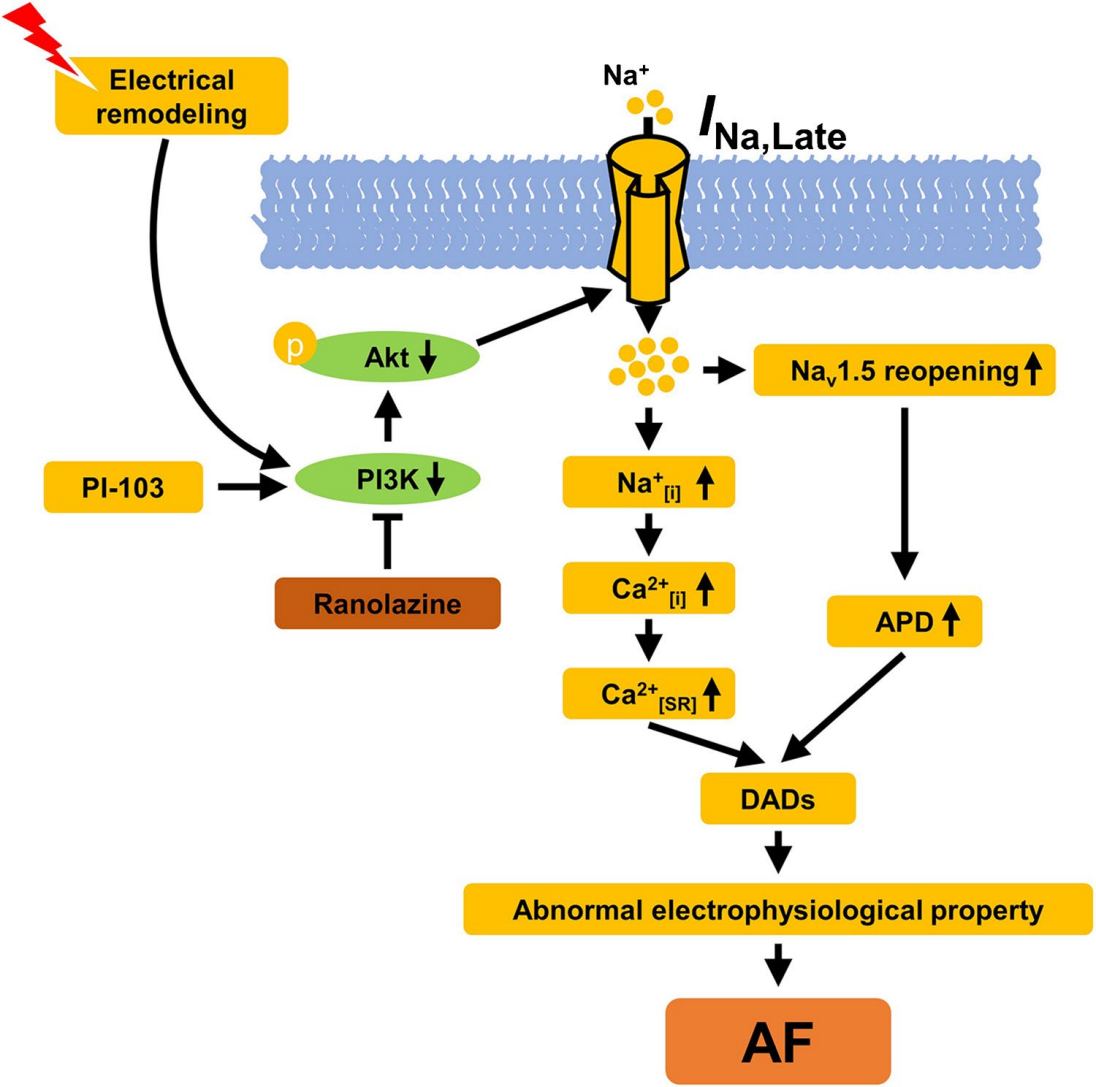
Scheme to demonstrate the mechanism for AF after *I*_Na,Late_ inhibition in tachypacing-induced HL-1 myocytes. Tachypacing or PI3K inhibition reduces PI3K p110α and pAkt expression, which further increases *I*_Na,Late_, and those detrimental effects are recovered by ranolazine treatment, which also reduces the accumulation of Na^+^ and Ca^2+^ and thereby prevents AF

In this study, we demonstrated that inhibiting *I*_Na,Late_ reversed abnormal Ca^2+^ handling and AP (prolonged APD and DADs) in tachypacing-induced HL-1 myocytes and that this recovery effect is related to the enhancement of PI3K/Akt signaling. Intriguingly, PI3K inhibition produced marked increases in *I*_Na,Late_ and subsequent contributions to aberrant AP morphology that were recovered by exposure to the *I*_Na,Late_ blocker ranolazine, suggesting that PI3K/Akt signaling plays a beneficial role in regulating pathogenic *I*_Na,Late_ and alterations in electrical remodeling. Together, these findings provide new insight into the potential role of the PI3K/Akt pathway in *I*_Na,Late_ and indicate that such regulation is critical to the treatment of AF (Fig. 8).

Na_v_1.5 mutations appear to play a pivotal role in the management of myocyte remodeling in AF. In particular, dysregulation of *I*_Na_ amplitude and duration contributes to incidence-triggered activity and prolonged AP and is related to severe AF [23, 24, 30]. Reductions in *I*_Na,Peak_ have been observed in arrhythmic disorders such as Brugada syndrome, long QT syndrome, and AF [10, 11, 28]. This effect further increases *I*_Na,Late_ by altering biophysical properties [19]. Thus, the combined gain and loss of *I*_Na_ function contributes to arrhythmic activity [4]. Previous studies reported reduced *I*_Na,Peak_ and enhanced *I*_Na,Late_ with biophysical defects in activation and inactivation in SCAN5A 1472del mutations of tsA201 cells [10]. Moreover, Lebek et al. [19] demonstrated that a negative shift in steady-state inactivation decreased *I*_Na,Peak_ and increased *I*_Na,Late_ while causing no difference in the activation curves of atrial myocytes in patients with sleep-disordered breathing. Although previous findings in clinical and experimental studies reported the dysregulation of *I*_Na_ in arrhythmia, the underlying mechanism and potential therapeutic options remain unclear. To clarify the pathological role of *I*_Na_ regulation in AF and the effect of *I*_Na,Late_ inhibition on *I*_Na_, we examined both *I*_Na,Peak_ and *I*_Na,Late_ amplitudes in tachypacing-induced HL-1 myocytes. In those cells, we observed decreased *I*_Na,Peak_ and increased *I*_Na,Late_, along with decreased kinetics of activation and inactivation and a depolarized shift rightward compared with non-paced HL-1 myocytes. Importantly, the consequences of pathological remodeling were all significantly reversed by *I*_Na,Late_ inhibition. Moreover, *I*_Na,Late_ inhibition recovered irregular Ca^2+^ handling and AP morphology. These results identify *I*_Na,Late_ as an efficient modulator of *I*_Na_ function and changes in electrical remodeling that could be used to prevent arrhythmic effects [15].

Importantly, a sustained increase in *I*_Na,Late_ is also a contributor to rising intracellular Na^+^, which induces intracellular Ca^2+^ accumulation and ultimately induces DADs and severe AF [4, 32]. We have demonstrated that increasing *I*_Na,Late_ contributes to Ca^2+^ dysregulation and the incidence of DADs, and those detrimental effects were abolished by *I*_Na,Late_ inhibition, suggesting that *I*_Na,Late_ could also be crucial in the regulation of Ca^2+^ handling. Of note, excessive intracellular Na^+^ overload caused by increasing *I*_Na,Late_ could be a result of reduced PI3K signaling, which leads to further intracellular Ca^2+^ overload [34]. Although we did not observe that Ca^2+^ signaling had any effect on PI3K signaling, it is possible that PI3K-mediated *I*_Na,Late_ regulation is responsible, at least in part, for alternating irregular Ca^2+^ release with basal Ca^2+^ levels. Further studies will be needed to examine the role of PI3K signaling in Ca^2+^ signaling under AF conditions.

Reducing or inhibiting PI3K/Akt signaling induces an increase in *I*_Na,Late_ through alterations of the *I*_Na_ gating property and exacerbates the cardiac remodeling related to arrhythmias [34]. Therefore, modulation of PI3K/Akt signaling could be informative in regulating pathogenic *I*_Na,Late_ and treating AF. Our data support the hypothesis that PI3K/Akt signaling plays an important role in *I*_Na,Late_ regulation for the management of AF in tachypacing-induced HL-1 myocytes. That loss of PI3K signaling significantly enhanced *I*_Na,Late_ due to decreased kinetics of activation and inactivation, which further contributed to prolonged APD and DADs, which are related to AF. Of note, PI3K inhibition is implicated as a consequence of *I*_Na,Late_ dysfunction and subsequent arrhythmia [21]. In fact, PI3K inhibition for 2 h by means of PI-103 or nilotinib resulted in increases in *I*_Na,Late_ in canine ventricular myocytes. In addition, Yang et al. [33] demonstrated that inhibiting the PI3Kɑ-specific subunit caused increases in *I*_Na,Late_ and further contributed to prolonged APD and triggered activity. Our results demonstrate that PI3K inhibition leads to increased *I*_Na,Late_, aberrant APD, and the occurrence of DADs, which are all abolished by *I*_Na,Late_ inhibition and the consequent upregulation of PI3K 110α and pAkt. Moreover, we discovered that a reduction in *I*_Na,Late_ occurred after application of PIP3 in tachypacing- or Akt-inhibited HL-1 myocytes. This suggests that a reduction in PI3K/Akt signaling contributes to the enhancement of *I*_Na,Late_ and the dysregulation of *I*_Na_ open probability, which can lead to further accumulation of intracellular Na^+^ under AF conditions.

Our study has several limitations. First, the constructed cellular AF model induced myocyte remodeling and thus might not fully represent the environments and circumstances of AF in vivo. Because upregulation of the PI3K/Akt pathway is involved in oncogenesis, appropriate dosages and the potential side effects of using ranolazine to treat AF need to be further investigated [27]. Second, our animal experiment revealed prolongation of the QT interval with a slight increase in RR interval (decreased heart rate) on PI3K inhibition. QT interval prolongation is a major determinant of the risk of arrhythmia and is caused by electrical repolarization abnormalities [1, 26]. Although we did not examine the inducibility of atrial arrhythmia, it is possible that prolongation of QT interval by PI3K inhibition is due to pathogenic *I*_Na,Late_ regulation as well as an increase in APD.

In conclusion, these findings highlight the role of PI3K/Akt signaling in pathogenic *I*_Na,Late_ and suggest ways that it might be used against AF. This study supports the identification of PI3K/Akt as an effective factor for regulating pathogenic *I*_Na,Late_ and preventing AF caused by aberrant electrical remodeling.
